# Case of Gangrenous Stomach, with Dysphagia, from Lightning

**Published:** 1800

**Authors:** Patrick Paterson

**Affiliations:** Surgeon of the Twenty-Ninth Regiment of Light Dragoons.


### VI. *Caſe of Gangrenous Stomach, with Dyſphagia, from Lightning. Communicated in a Letter to Dr.* Simmons, *by Mr.* Patrick Paterſon, *Surgeon of the Twenty-ninth Regiment of Light Dragoons.*

NATHANIEL BAILEY, a recruit of the twenty-ninth regiment of Light Dragoons, thirty-five years of age, of a corpulent habit of body, and rather diſpoſed to obeſity, applied to me on Friday the 30th of June, on account of failure in his ſight. On examining his eyes, I found the pupils much dilated, and the iris of each but very little ſenſible to the impreſſion of light. He could not read the largeſt print however near him; and objects at a diſtance put on a confuſed and indefinite form, and ſeemed of a much larger ſize than natural. He at the ſame time complained of ſome uneaſineſs, and ſenſe of fulneſs, in the epigaſtric region; and on attempting to ſwallow, either ſolids or fluids, he felt ſome reſiſtance to their deſcent, high up in the throat. The uneaſineſs, however, in the region of the ſtomach,

ſtomach, was not increaſed on preſſure, nor was it accompanied, in any degree, with either nauſea or vomiting. He had alſo ſlight head ach, which was confined chiefly to the fore part; his pulſe was natural; his belly bound; and appetite bad.

He dated the origin of his complaints from Wedneſday the 28th of June, when travelling on the top of a ſtage coach, from Perſhore to this place, he had ſenſibly felt a flaſh of lightning affect his ſight, and ſoon after experienced ſome uneaſineſs in the ſtomach. On interrogating him relative to this circumſtance, he ſaid, he felt himſelf ſomewhat ſhocked at the time the lightning dazzled his ſight, but could not poſitively ſay, whether it proceeded from the lightning, or from an accidental movement of the carriage, ſo tranſient was the ſenſation. Being apprehenſive that the affection of the eyes was of a longer ſtanding than he was willing to acknowledge, I directed my attention chiefly to the ſtate of the ſtomach and bowels, and gave him a bolus, containing eight grains of calomel, which he ſwallowed with ſome difficulty; and I ſaw him no more till early the next morning.

Saturday, July 1.—The bolus had not operated; the ſenſe of fulneſs and uneaſineſs about the ſtomach was conſiderably increaſed; and likewiſe the difficulty of digeſtion was greater. What he attempted to paſs into the ſtomach, was, after a ſhort effort, returned through the noſe, with a kind of convulſive ejectment, without having penetrated beyond the pharynx. This, however, was not univerſally the caſe; for if any thing paſſed this point, it did not meet with any further reſiſtance, but paſſed into the ſtomach. He now began to articulate ſomewhat indiſtinctly, like one who had a ſlight degree of the croup, and complained of dryneſs of the tongue and fauces, without thirſt; but from the examination of thoſe parts, nothing unnatural or diſeaſed could be diſcovered. He alſo thought his hearing impaired, but this was not very evident. Coſtiveneſs ſtill prevailing, I gave him two ſcruples of powdered jalap, with the ſame quantity of cream of tartar, which he got down with ſome difficulty, a ſmall quantity regurgitating by the noſe, after every exertion to ſwallow. The ſtate of his eyes remained as yeſterday. His pulſe and ſkin were both natural.

 I viſited

I viſited him in the evening, and found his complaints much worſe. His breathing was ſeemingly affected, eſpecially when he raiſed himſelf up, but of this he did not complain; and this apparent dyſpnœa ſoon went off, after his reſuming the recumbent poſition. Reſpiration was, however, attended with a ſtridulous kind of noiſe, and I apprehended there might be ſome degree of inflammation of the larynx and its vicinity, which gave riſe to this, and alſo to the difficulty of ſwallowing. This was the only reaſon I had to ſuppoſe that blood-letting might be of ſome ſervice, but I proceeded altogether empirically, not being acquainted, ſcarcely in any degree, with the real nature of the caſe, from the unaccountable combination and appearance of the ſymptoms. I determined, however, on bleeding him, and took from his arm ten ounces of blood; when deliquium ſupervening, obliged me to deſiſt. As the purgative he took in the morning had produced no effect, I preſcribed two injections of ſalt and water with oil; the firſt operated ſlighty, but the ſecond brought off a conſiderable quantity of fæces, hard, but of a natural appearance in other reſpects; the greater part of which he paſſed in bed, with little previous notice notice or defire to go to ftool. His fight was more defective than hitherto; his articulation worfe; his urine high coloured, and in fmall quantity. His pulfe and fkin were natural; his tongue was dry, but he had no thirft. I left him very little fatisfied with what I had done for him, and from my ignorance of his cafe, could not fuggeft any means of relief that had the fhadow of reafon to recommend it.

Sunday, July 2.—All the fymptoms were aggravated. The uneafinefs (for it did not amount to pain) about the fcrobiculus cordis, was the chief fubject of complaint, and my patient expreffed much uneafinefs "that no-"thing," as he faid, "could be made to pafs "through him." As the evacuation by means of the glyfters ferved to empty only a fmall portion of the inteftines, I was ftill of opinion, that purgatives might relieve him, and exhibited, with that hope, an ounce and a half of caftor oil, with half an ounce of tincture of fenna; this he fwallowed, with fome difficulty, about nine in the morning; and in the mean time I went to confult with Dr. Milne, one of the phyficians to the Infirmary of this place, conceiving my patient to be in imminent danger. Dr. Milne was ſo obliging as to viſit him with me, and after conſidering the caſe for ſome time, he found it not to agree with any other he had ever ſeen, either viewing the whole of the ſymptoms collectively, or conſidering ſome of them apart. On Dr. Milne's aſking him whether he had taken any thing into his ſtomach, that he ſuppoſed might have diſagreed with him, he replied, that, on Wedneſday (the ſame day he felt his ſight affected by the lightning) being very hungry, he had eaten a very large quantity of cheeſe for his breakfaſt, and which he thought had never paſſed by ſtool. Dr. Milne, I believe, partly on the ſuppoſition that ſomething was retained by the ſtomach, of which it was unable to free itſelf by any natural efforts, and partly with a view to excite the action of the alimentary canal, which ſeemed in a very torpid ſtate, propoſed to try an emetic. This was agreed to, from a conviction that there could be no inflammation of the ſtomach, as there was a total abſence of all the ſymptoms of gaſtritis. It was alſo ſuggeſted to apply a bliſtering plaiſter afterwards to the region of the ſtomach, or to the external fauces; and the patient, as a part of of the ſtimulating plan, was directed to chew ſmall pieces of mezereon. The emetic thought of as the moſt quick in its operation, and as ſafe as any other, was vitriolated zinc. At firſt I exhibited this in a doſe of ten grains, but finding that in leſs than an hour no effect whatever was produced from this quantity, I gave him twenty grains more. This, however, was followed with as little effect as the other doſe; and being anxious to induce vomiting at all events, but not thinking it prudent to perſiſt in the uſe of a metallic preparation, I mixed up thirty grains of powdered ipecacuanha, but my patient obſtinately refuſed to ſwallow it, and perſiſted in this reſolution.

Suppoſing that ſome part of the difficulty of deglutition might depend on ſome local affection of the pharynx or the ſurrounding parts, I applied a bliſtering plaſter to the throat, and took my leave of the unhappy man with as little ſatisfaction as ever. He had occaſionally got down ſome ſoup; but not relying on this ſcanty ſupport, I left directions that ſtrong mutton broth might be frequently thrown up by way of glyſter.

Monday, July 3.—This day brought with it an aggravation of all the ſymptoms, and the man's ſtrength began to flag apace. Deglutition was extremely difficult; articulation very indiftinct; vifion ftill more impaired; the pulfe fmall but not quick; he had had no ftool, and had made hardly any urine: but his intellects were unimpaired. I again waited on Dr. Milne, and having given him fome defcription of the fituation of the patient, he ftill wifhed to try the effects of vomiting, and advifed muftard as an emetic, quick in its operation, and at the fame time, from its ftimulating and pungent nature, well adapted to this cafe, which difcovered fo inexcitable a degree of torpor in the whole of the alimentary canal. I accordingly mixed up fome ftrong frefh powdered muftard with water, of as thick a confiftence as the man could fwallow it, and with much difficulty got him to take two large fpoonfuls, at a fhort interval between each. It gave him moft uneafinefs when paffing the fauces, but once paffed, it went down with tolerable eafe; I gave it him carefully with my own hand, and had the fatisfaction to find that no part of it returned by the nofe. It produced, however, no further effect than confiderable uneafinefs in the throat, and fhort convulfion like efforts, as if he were about to vomit, which foon went off. He could not be perfuaded perſuaded to uſe any more of the muſtard, but conſented to take a draught containing half a drachm of powdered ipecacuanha; but after waiting an hour, I had the mortification to find no effect produced by it. I now gave up all hopes, and concluded there muſt be ſome mortal affection of the ſtomach, which had utterly deprived it of the power of being ever again called into action. Dr. Milne ſaw the man in the courſe of the forenoon, and gave a fatal prognoſtic. He accordingly died about four o'clock in the afternoon, without any ſtruggle or any apparent pain.

Next day, in the preſence of Dr. Milne and Meſſieurs Jeffreys and Cole, Surgeons, of this place, I opened the cavities of the thorax and abdomen; and the viſcera of both, at firſt view, preſented a natural and healthy appearance. On proſecuting the diſſection, and tracing the alimentary canal from the beginning of the pharynx downwards to the ſtomach, we came to morbid appearances ſufficient to have produced death, however difficult it may be to reconcile them with the ſymptoms that took place during the patient's illneſs. The pharynx and oeſophagus had no viſible mark of diſeaſe; but on cutting into the ſtomach a very large extent of it was found in a highly gangrenous ſtate. This we had reaſon to expect, from the inflamed and livid appearance externally. The inflammation, however, was evidently not that of great action, and the liver was ſtreaked with a dingey yellow. The gangrene commencing near the cardia, extended over the whole of the ſtomach to within two or three inches of the pylorus, which portion was perfectly ſound. The ſtomach contained only the medicines he had taken that and the preceding day, viz. the caſtor oil and tincture of ſenna, the muſtard, ipecacuanha, and a ſmall quantity of green vegetable matter he had taken in ſome ſoup. A portion of the meſocolon, of about the ſize of half a crown, was of a high red colour, and of a very fine delicate membranous texture. The whole of the meſentery and meſocolon was thickly filled with fat, excepting at this very point. This portion was cut out, and being expoſed for ſome time to the air, it loſt entirely its florid hue, and became quite colourleſs and tranſparent. Theſe were the only morbid appearances obſerved in the viſcera. The head was not opened. The inteſtines contained a quantity of hard feculent matter, but did not exhibit any diſeaſed appearance whatever.

How far lightning may have been the occaſional cauſe of the diſeaſe, or whether the extinction of the nervous energy in the ſtomach and parts connected with it, ought to be conſidered as the proximate one, I ſhall leave to the inveſtigation of others, whoſe reſearches lead them to inquire into the relative nature of the electric fluid and the nervous influence; and ſhall only take notice, that the retention of animal heat, as well as the fluidity of the blood, after death, was very remarkable, though putrefaction had taken place in various parts of the body, and that accompanied with the moſt diſagreeable fetor.

*Worceſter, July 22, 1797.*

